# Use of Electronic Health Records and Quality of Ambulatory Healthcare

**DOI:** 10.7759/cureus.30343

**Published:** 2022-10-16

**Authors:** Duaa Alammari, Jim E Banta, Huma Shah, Ellen Reibling, Shrenik Talsania

**Affiliations:** 1 Health System Management, King Saud Bin Abdulaziz University for Health Sciences, Riyadh, SAU; 2 Public Health, King Abdullah International Medical Research Center, Riyadh, SAU; 3 School of Public Health, Loma Linda University, Loma Linda, USA; 4 Department of Emergency Medicine, Loma Linda University, Loma Linda, USA

**Keywords:** office-based physician, prevention, screening, ambulatory care setting, quality measures, electronic health records

## Abstract

Background

This study aimed to measure the association between electronic health record (EHR) use and quality measures in ambulatory healthcare.

Methodology

A quantitative, retrospective, cross-sectional design was used by examining secondary data from the 2015-2016 National Ambulatory Medical Care Survey. The relationship between EHR use and seven quality measures was examined using the Donabedian model as a framework. Quality measures included (a) diabetes measures, (b) obesity measures, (c) blood pressure screening, (d) depression screening, and (e) breast cancer screening. A total of 37,290 office visits were included, representing 817 million national office visits. For each of the quality measures, we determined the (a) associations using unadjusted and adjusted regression models based on subsets of the sample that met the inclusion criteria for quality measures; and (b) the changes in the area under the curve (AUC).

Results

Approximately 75% of office visits fulfilled all EHR use. Positive associations were found between EHR use and better quality for the following three out of seven measures: higher odds of screening for obesity (odds ratio (OR) = 2.2; p = <0.0001), blood pressure (OR = 2.5; p = <0.0001), and breast cancer (OR = 1.8; p = 0.0166). Receiver operating curve results showed the highest gain in the AUC for process-grouped measures. Hence, it was considered to be a strong predictor for all quality measures.

Conclusions

Evidence showed improvement in some quality measures (screening for obesity, blood pressure, and breast cancer). Common and standardized health processes were more likely to be completed and recorded than others. Future policies concerning health information technology can shift the focus from improving EHR use to enhancing patient and quality outcomes. Further research is needed to identify circumstances where quality is improved.

## Introduction

In the United States, there has been an increasing interest in health information technology (HIT). For example, electronic health records (EHRs) were mandated after 2014 as part of larger healthcare reform, with the goal of improving multiple aspects of care, including quality, safety, and cost [1,2]. EHR adoption increased rapidly between 2008 and 2015 from 42% to 89% among office-based physicians [3].

Healthcare providers are now required to report quality metrics called Clinical Quality Measures (CQMs) to the Centers for Medicare and Medicaid Services (CMS) to receive incentives and avoid penalties under the EHR Incentive Programs [4,5]. CQMs are tools that help measure and track the quality of healthcare service delivery [1].

Currently, billions of dollars are spent on adopting EHR systems and fulfilling reporting requirements for quality measures [6,7]. Yet, the literature presents mixed results regarding the relationship between EHR use and quality measures. Multiple studies have indicated that EHRs have clear advantages over paper records, such as accessibility, availability, and portability [8-11]. A systematic review concluded that overall EHR use could improve quality indicators due to better adherence to medical guidelines and reduced inappropriate care [12]. Kern et al. (2013) reported a positive correlation between physicians’ use of EHRs and ambulatory care quality in a community-based setting. They found that four out of nine individual measures showed significant improvements and that EHR use generally was positively correlated with better quality of care compared to paper records [13].

However, EHRs do not consistently deliver positive impacts on quality indicators [14,15]. A comprehensive scoping review presented mixed results after examining the relationship between HIT and quality of care in the federal healthcare system [16]. Furthermore, clinical decision support systems seem to improve physicians’ adherence to guidelines, though evidence does not indicate a statistically significant association [16]. Another systematic review assessed the impact of EHR use on structure, process, and outcomes by applying the Donabedian model in primary care [14]. The study reported that EHR use positively impacted structure and process compared to paper records [14] and was associated with improved quality measures, such as rates of screening measures for blood pressure, depression, and breast cancer [13,17,18].

EHR use has been linked with multiple beneficial diabetes-related outcomes when compared to paper records [19]. For instance, EHR use was linked to higher rates of HbA1c testing among patients with diabetes [13]. However, other studies did not find that consistent use of EHRs improved the quality of diabetes care [20].

Mixed results have been reported for obesity screening and prevention measures. Tracking obesity measures improved after the adoption of EHRs in a small practice in north Texas [21]. Likewise, there are higher odds of obesity screening (body mass index (BMI) documentation) with EHR use [22]; however, another study indicated a lack of BMI documentation in EHR records [23]. Romano and Stafford found that using EHRs was linked to higher rates of obesity counseling [24]; however, other studies found insignificant differences in exercise and dieting rates among high-risk adults [22,25].

Given these conflicting findings, our goal was to examine the relationship between EHR use and seven quality measures in ambulatory healthcare using elements of the Donabedian model. The Donabedian model has been used elsewhere to assess the quality of care in health services [26,27]. This study only examined structure and process measures but not outcomes [26,27].

## Materials and methods

Study design and sample

This study used a quantitative, retrospective, cross-sectional design. Secondary data were obtained from the 2015-2016 National Ambulatory Medical Care Survey (NAMCS) [28]. NAMCS is a national probability sample survey based on a sample of visits to non-federally employed office-based physicians in the United States [28]. This study received Institutional Review Board exemption due to de-identified medical records as the primary data source.

The response rate for physicians having at least one sampled visit was 30% for 2015 and 39% for 2016 [28]. Researchers restricted the analytic sample using the following criteria: (a) fulfilling the inclusion criteria of at least one of the seven quality measures, and (b) patients aged 12 years or older. Visit records with missing EHR use status were excluded (46 visits). A total of 37,290 office visits met the inclusion criteria. However, each quality measure had a unique population with a defined age parameter (see Table 1).

**Table 1:** Quality measures’ description, sample size, and percentages of patient visits to ambulatory office-based physicians from NAMCS 2015-2016 survey data. Eligible professional quality reporting criteria for 2019 CMS (numerator, denominator (population) for eligible professionals). *: CQM has two levels: screening and education for eligible patients. NAMCS: National Ambulatory Medical Care Survey; CQM: Clinical Quality Measure; BMI: body mass index

Quality measures	Numerator	Denominator	Sample size	Visits with test n (%)
Diabetes eye exam screening	Patients with an eye screening exam	Patients 18–75 years of age with diabetes	2,087	577 (27.7)
Diabetes HbA1c screening	Patients with documented HbA1c			353 (16.9)
Obesity screening and education*	Patients with documented BMI	Patients 18 years and older	35,612	14,533 (40.8)
	Patients with a documented follow-up plan for weight management	Patients 18 years and older with a BMI of 25 or more	12,260	2,273 (18.5)
Breast cancer screening	Women with one or more mammograms documented	Women patients 51–74 years of age	9,210	360 (3.9)
Depression screening	Patients screened for depression	Patients aged 12 years and older	37,290	1,327 (3.6)
Blood pressure screening	Patients with a documented blood pressure measurement	Patients 18 years and older	35,612	21,644 (60.8)

Quality measures

Proxy quality measures were inspired by literature and the CQMs used in the CMS Incentive Programs [29]. Six screening measures and one education/counseling measure focused on the following five different conditions: (a) diabetes ((i) eye exam screening, (ii) HbA1c testing); (b) obesity ((i) BMI measurement, (ii) education); (c) blood pressure screening; (d) depression screening; and (e) breast cancer screening (see Table 1). All quality measures were dichotomized to indicate whether appropriate care was provided.

All quality measures were tested using a single variable except for obesity education and blood pressure screening. Availability of both systolic and diastolic readings was used for blood pressure screening, and delivery of at least one of the three education/counseling services (diet/nutrition, exercise, and weight reduction counseling) was used for obesity education. Obesity screening was measured using BMI regardless of the recorded measurement.

Electronic health records

The level of EHR use was recorded as Yes for all EHR use and No for partial/no EHR use.

Donabedian Model (Structure and Process)

Structure included (a) office region (Midwest, Northeast, South, West); (b) location (metropolitan, non-metropolitan); (c) office type (private solo/group practice, others); and (d) EHR system replacement plans (yes, no, unknown/maybe).

Visit process included (a) primary care provider (yes, no); (b) physician specialty (obstetrics-gynecology, ophthalmology, psychiatry, others); (c) visit duration (1-29 minutes, 30 minutes+); (d) patient referred to visit (yes, no, N/A); (e) new patient (yes, no); (f) number of past visits (0, 1-5, 6+); and (g) payment for visit (private, Medicare/state or Medicaid, others).

To fulfill the statistically minimum number of subjects in each cell, some answer categories were collapsed, such as “physician specialty,” “patient referred to visit,” and “payment for visit.” Missing data were grouped with “other” categories.

Patient Demographics

Demographics included (a) age (recoded as 12-17 years, 18-49 years, 50-64 years, 65 years or over); (b) gender (male, female); (c) race (white, others); and (d) ethnicity (Hispanic, non-Hispanic).

Statistical analysis

Descriptive statistics (chi-square) and multivariate logistic regressions were used to examine the association between EHR use and quality measures. The Donabedian model was used as a conceptual framework in the analysis to explore the organizational structure and process components related to EHR use.

Researchers conducted two different analytical approaches. First, unadjusted and adjusted regression models were for each quality measure. Covariates included structure, process, and demographics. A p-value of less than 0.05 was used to determine statistical significance. Sample sizes for regression analysis ranged from 2,087 to 37,290 office visits depending on the quality measure’s inclusion criteria.

Second, researchers used a receiver operating curve (ROC) approach by examining changes in the area under the curve (AUC). Covariates were grouped into three categories, namely, structure, process, and demographics. Starting with EHR use, consecutively adding elements of the Donabedian model (structure and process), then demographics for a total of four models for each quality measure [30]. All analyses took into consideration the complex survey design and were performed using SAS software version 9.4.

## Results

This study included 37,290 office visits, representing an annual estimate of 817.6 million national office visits to ambulatory care physician offices. Office visits were stratified by EHR use status; all EHRs were used in 75% of visits, and partial/no EHRs in 25% of visits. Descriptive statistics showed that most patients were white or non-Hispanic/Latino (see Table 2).

**Table 2:** Authors’ analysis of data from NAMCS 2015-2016 survey. Weighted office visits’ characteristics among ambulatory physician offices (n = 37,290/N = 817,637,119) patient office visits. The numbers presented in the table are percentages. *: p-value = 0.05; **: p-value = 0.005; =: unknown was grouped with other categories. NAMCS: National Ambulatory Medical Care Survey; EHR: electronic health record

EHR use	Yes, all EHR	None or partial EHR use	P-value
Total	28,043 (75.2)	9,247 (24.8)	
Patient demographics	%	%	
Age			0.2620
12–17 years	5.5	5.1	
18–49 years	33.8	35.7	
50–64 years	26.5	28.6	
65 years and over	34.2	30.6	
Gender			0.1093
Female	59.4	62.4	
Male	40.6	37.6	
Ethnicity			*0.0097
Non-Hispanic or Latino	87.6	81.3	
Hispanic or Latino	12.4	18.7	
Race			*0.0178
White	69.9	62.1	
Other	30.1	37.9	
Organizational structure			
Region			**0.0001
Northeast	16.5	31.1	
Midwest	21.3	15.0	
South	38.2	27.7	
West	24.0	26.2	
Location			0.1038
Urban	91.8	95.2	
Rural	8.2	4.8	
Type of office			0.1823
Private/group practice	91.0	94.0	
Other	9.0	6.0	
System replacement plans			*0.0079
Yes	10.9	15.0	
No	80.7	68.3	
Unknown/maybe=	8.4	16.7	
Visit process			
Primary care provider			**0.0038
Yes	41.3	28.3	
No=	58.7	71.6	
Specialty			**<0.0001>
Other	81.9	68.0	
OBGYN	9.3	9.8	
Ophthalmology	5.1	9.1	
Psychiatry	3.7	13.1	
Referred for visit			**0.0017
Yes	18.4	19.8	
No	31.4	42.1	
Unknown/Not applicable=	50.3	38.1	
New patient			**0.0020
Yes	16.8	12.9	
No	83.2	87.1	
Number of past visits			**<0.0001>
1–5	56.6	52.1	
6+	20.1	27.4	
No visit	6.5	7.6	
Time spent with MD			
0–29 minutes	75.9	64.4	**<0.0001>
30+ minutes	24.1	35.6	
Payment for visit			
Medicare/State/Medicaid	40.4	41.3	*0.0491
Private insurance	48.7	43.3	
Other=	10.9	15.4	

Comparing visits in both EHR use groups, researchers found that the northeast region had the highest rate of visits with partial/no EHR use (31%), while the south region had the highest rate of visits with all EHR use (38%). Psychiatry had higher visit rates with partial/no EHR use (13%) compared to visits with all EHR use (4%). More than one-third of patients (36%) spent more time with their physician when partial/no EHR was used compared to all EHR use (24%) (see Table 2).

Table 3 and Table 4 present unadjusted and adjusted regression models. Researchers found statistically significant positive associations between EHR use and three quality measures, namely, higher odds of obesity screening, breast cancer screening, and blood pressure screening with all EHR use.

**Table 3:** Analysis of data from NAMCS 2015-2016 survey. Unadjusted and adjusted regression models for EHR use and seven quality measures among patient visits to office-based physicians. *: p-value = 0.05; **: p-value = 0.005; +: borderline significant. We controlled for (1) system replacement plans as a structure measure and (2) survey year process measure in the logistic regression but OR and p-values are not presented in the table. NS: non-sufficient sample size for a valid OR. NAMCS: National Ambulatory Medical Care Survey; EHR: electronic health record; OR: odds ratio

Quality measures	Diabetic eye care		Diabetic HbA1C		Obesity screening		Obesity education	
EHR use	OR	P-value	OR	P-value	OR	P-value	OR	P-value
None or partial (reference)								
Yes, all electronic (unadjusted)	0.49	0.0514+	1.69	0.1259	2.58	<0.0001>	0.81	0.4484
Yes, all electronic	0.79	0.5149	1.43	0.3606	2.27	<0.0001>	0.90	0.6435
Structure								
Region								
Midwest (reference)								
Northeast	0.90	0.8401	1.26	0.4650	1.24	0.2673	1.12	0.6206
South	1.49	0.4030	1.21	0.4793	1.20	0.2878	1.20	0.4233
West	1.37	0.4493	0.65	0.2465	0.94	0.7935	0.81	0.4689
Location								
Urban (reference)								
Rural	0.69	0.5170	NS	NS	1.18	0.4445	0.84	0.5796
Type of office								
Solo/group practice (reference)								
Other	1.52	0.4812	1.37	0.2519	0.77	0.3134	0.94	0.8407
Process								
Primary care provider								
No (reference)								
Yes	1.05	0.8915	5.14	0.0001**	2.81	<0.0001>	1.73	0.0474*
Specialty								
Other (reference)								
OBGYN	NS	NS	NS	NS	0.38	<0.0001>	0.71	0.3079
Ophthalmology	169.95	< .0001>	1.06	0.9175	0.07	<0.0001>	NS	NS
Psychiatry	NS	NS	NS	NS	0.40	<0.0001>	0.51	0.1085
Visit duration								
1–29 minutes (reference)								
30+ minutes	1.00	0.9794	1.65	0.0105*	1.40	0.0006**	1.14	0.3037
Referral								
No (reference)								
Yes	3.67	0.0442*	NS	NS	0.94	0.6892	0.79	0.4392
Not applicable	3.27	0.0325*	NS	NS	1.05	0.7537	0.98	0.9346
New patient								
Yes (reference)								
No	0.84	0.7989	NS	NS	0.73	0.0033**	1.40	0.0284*
Number of past visits								
1–5 (reference)								
6+	0.51	0.0095*	NS	NS	0.74	0.0015**	0.96	0.7742
No visits	1.69	0.2034	NS	NS	1.38	0.0023**	1.05	0.6837
Payment for visit								
Private insurance (reference)								
Medicare/state-Medicaid	0.98	0.9538	NS	NS	1.08	0.3602	0.94	0.5803
Other	0.21	0.0002**	NS	NS	0.87	0.4293	0.94	0.7435
Demographics								
Age (years)								
12–17 years	0.49	0.0099*	0.03	< .0001>	0.43	<0.0001>	0.59	0.0017**
18–49 years	0.56	0.1276	0.94	0.8580	0.63	<0.0001>	0.80	0.0245*
50–64 years (reference)								
65 years and over	0.89	0.6818	0.78	0.1831	0.80	0.0052*	0.90	0.3591
Gender								
Male (reference)								
Female	0.74	0.1617	1.27	0.2435	1.01	0.7298	1.08	0.3431
Race								
White (reference)								
Other	0.71	0.3539	1.47	0.2411	1.29	0.0632	1.21	0.2303
Ethnicity								
Non-Hispanic/Latino (reference)								
Hispanic or Latino	2.18	0.0655	0.62	0.2433	1.03	0.8627	1.73	0.0263*

**Table 4:** Analysis of data from NAMCS 2015-2016 survey. Unadjusted and adjusted regression models for EHR use and seven quality measures among patient visit to office-based physicians (continued). *: p-value = 0.05; **: p-value = 0.005. We controlled for (1) system replacement plans as a structure measure and (2) survey year as a process measure in the logistic regression, but OR and p-values are not presented in the table. NS: Non-sufficient sample size for the valid odds ratio. NAMCS: National Ambulatory Medical Care Survey; EHR: electronic health record; OR: odds ratio

Quality measures	Breast cancer screening		Depression screening		Blood pressure screening	
EHR use	OR	P-value	OR	P-value	OR	P-value
None or partial (reference)						
Yes, all electronic (unadjusted)	2.17	0.0062*	1.03	0.9235	2.84	<0.0001>
Yes, all electronic	1.76	0.0166*	1.00	0.9798	2.54	<0.0001>
Structure						
Region						
Midwest (reference)						
Northeast	0.68	0.2831	0.70	0.3222	0.87	0.5192
South	0.92	0.7731	0.65	0.2094	1.09	0.6202
West	0.79	0.5415	0.39	0.0021**	0.77	0.1849
Location						
Urban (reference)						
Rural	0.67	0.3714	0.61	0.1954	1.36	0.3168
Type of office						
Solo/group practice (reference)						
Other	1.06	0.8376	2.34	0.0806	1.68	0.0237
Process						
Primary care provider						
No (reference)						
Yes	3.28	0.0033**	5.26	<0.0001>	10.44	<0.0001>
Specialty						
Other (reference)						
OBGYN	12.02	<0.0001>	1.23	0.5353	7.56	<0.0001>
Ophthalmology	NS	NS	0.52	0.2584	0.08	<0.0001>
Psychiatry	NS	NS	7.94	<0.0001>	0.40	0.0001**
Visit duration						
1–29 minutes (reference)						
30+ minutes	1.03	0.8610	1.20	0.2731	1.61	<0.0001>
Referral						
No (reference)						
Yes	0.49	0.1167	1.00	0.9997	0.93	0.6333
Not applicable	0.97	0.9427	0.96	0.9170	0.86	0.4150
New patient						
Yes (reference)						
No	0.50	0.0140*	0.64	0.0068*	0.92	0.4152
Number of past visits						
1–5 (reference)						
6+	0.68	0.1465	0.89	0.4077	0.83	0.0873
No visits	3.01	<0.0001>	1.55	0.0551	0.90	0.3060
Payment for visit						
Private insurance (reference)						
Medicare/state-Medicaid	NS	NS	1.14	0.4024	1.23	0.0130*
Other	NS	NS	1.45	0.3505	0.87	0.4583
Demographics						
Age (years)						
12–17 years	-	-	0.98	0.9472	0.26	<0.0001>
18–49 years	-	-	1.17	0.2471	0.80	0.0022**
50–64 years (reference)						
65 years and over	1.02	0.8691	1.25	0.1559	0.95	0.5918
Gender						
Male (reference)						
Female	-	-	1.15	0.1835	1.07	0.2151
Race						
White (reference)						
Other	1.37	0.1375	0.86	0.5094	1.61	0.0019**
Ethnicity						
Non-Hispanic or Latino (reference)						
Hispanic or Latino	0.76	0.4083	1.14	0.6560	0.85	0.3534

Diabetes measures

An adjusted regression model did not find a significant association between EHR use and eye screening among diabetes patients, though the results were borderline significant (p = 0.0514) in the unadjusted model. Patients with diabetes were more likely to get eye screening if they were seen by an ophthalmologist (odds ratio (OR) = 169.95) or referred for a visit (OR = 3.7), but they were less likely to get screened if they were 12-17 years old, visited the facility more than six times, or if payment for the visit was unknown or patient pay.

There was no significant association between EHR use and diabetes HbA1c testing. Diabetic patients were more likely to get tested for HbA1c if they saw their primary care provider (OR = 5.1) or spent more than 30 minutes with the provider (OR = 1.7), but they were less likely to get tested for HbA1c if they were 12-17 years old (Table 3).

Obesity screening and education

Office visits with all EHR use were more likely to be screened for obesity when compared to visits with partial/no EHR use in both adjusted (OR = 2.2) and unadjusted (OR = 2.6), regression models.

Patients were more likely to be screened for obesity if they saw their primary care provider (OR = 2.8), spent more than 30 minutes with the provider (OR = 1.4), or had no previous visits (OR = 1.4). They were less likely to be screened for obesity if they were younger than 50 years of age or 65 years and over, saw specialists (OBGYN, ophthalmologist, or psychiatrist), were established patients at the facility, or had more than six visits.

There was no significant association between obesity education among overweight and obese patients in both adjusted and unadjusted models. Patients were more likely to receive obesity education if they were non-Hispanic or Latino (OR = 1.7), saw their primary care provider (OR = 1.7), and were established patients at the facility (OR = 1.4). They were less likely to receive obesity education if they were less than 50 years of age (Table 3).

Breast cancer screening

Females visiting offices with all EHR use were more likely to receive mammograms when compared to visits with partial/no EHR use for both adjusted (OR = 1.8) and unadjusted (OR = 2.2) regression models. Furthermore, females were more likely to receive mammograms if they saw their primary care provider (OR = 3.3), were seen by an OBGYN physician (OR = 12.0), and had no previous visits to the facility (OR = 3.0); however, they were less likely to receive mammograms if they were established patients at the facility (Table 4).

Depression screening

There was no significant association between EHR use and depression screening. Patients were more likely to be screened for depression if seen by their primary care provider (OR = 5.3) or psychiatrist (OR = 7.9); however, they were less likely to be screened for depression if they were seen in the west or were established patients (Table 4).

Blood pressure screening

Office visits with all EHR use had higher odds of blood pressure screening compared to visits with partial/no EHR in both adjusted (OR = 2.5) and unadjusted (OR = 2.8) regression models. Patients were more likely to get their blood pressure checked if they were non-white (OR = 1.6), saw their primary care provider (OR = 10.4) or an OBGYN physician (OR = 7.6), spent more than 30 minutes with the provider (OR = 1.6), and used Medicare/Medicaid for the visit (OR = 1.2); however, they were less likely to get their blood pressure checked if they were less than 50 years of age or seen by specialists (ophthalmologist or psychiatrist) (Table 4).

Table 5 shows the ROC analysis. The demographic group was not or a weak predictor across all quality measures with a gain in AUC ranging from 0.000 to 0.004. EHR use was a weak predictor for most quality measures except for obesity and blood pressure screening. Likewise, the structure group was a weak predictor except for diabetic HbA1c and depression screening. The process group was a strong predictor for all quality measures with a gain in AUC ranging from 0.117 to 0.280.

**Table 5:** Analysis of data from NAMCS 2015-2016 survey. ROC analysis of EHR use, structure components, process components, and demographics with all quality measures. *: The final result represents the full models shown in Table 3 and Table 4. NAMCS: National Ambulatory Medical Care Survey; ROC: receiver operator curve; AUC: area under the curve; EHR: electronic health record

Quality measures		No variables	Adding EHR use	Adding structure	Adding process	Adding demographics
Diabetic eye care	AUC	0.500	0.538	0.569	0.832	0.832
	Gain in AUC		0.038	0.031	0.263	0.000
	% of explained AUC		11.4	9.3	79.2	0.0
Diabetic HbA1C	AUC	0.500	0.513	0.585	0.831	0.831
	Gain in AUC		0.013	0.072	0.246	0.000
	% of explained AUC		3.9	21.8	74.3	0.0
Obesity screening	AUC	0.500	0.586	0.597	0.732	0.736
	Gain in AUC		0.086	0.011	0.135	0.004
	% of explained AUC		36.4	4.7	57.2	1.7
Obesity education	AUC	0.500	0.506	0.548	0.665	0.665
	Gain in AUC		0.006	0.042	0.117	0.000
	% of explained AUC		3.6	25.5	70.9	0.0
Breast cancer screening	AUC	0.500	0.538	0.560	0.840	0.841
	Gain in AUC		0.038	0.022	0.280	0.001
	% of explained AUC		11.1	6.5	82.1	0.3
Depression screening	AUC	0.500	0.513	0.583	0.722	0.722
	Gain in AUC		0.013	0.070	0.139	0.000
	% of explained AUC		5.9	31.5	62.6	0.0
Blood pressure screening	AUC	0.500	0.590	0.620	0.800	0.800
	Gain in AUC		0.090	0.030	0.180	0.000
	% of explained AUC		30.0	10.0	60.0	0.0

## Discussion

This study used NAMCS 2015-2016 survey data to examine associations between EHR use and seven quality measures. We found significant positive associations between EHR use and better quality for three quality measures. The adjusted and unadjusted regression models’ results were reasonably similar suggesting that adding additional structure, process, and demographic factors did not have a substantial effect on quality measures. However, the ROC analysis results showed a noteworthy increase in AUC after adding process measures.

The findings of this research are consistent with prior studies which suggested that EHR use was not always positively associated with improvements in quality measures [15,17]. This research updated and expanded upon the analysis by Hsiao et al. (2014), Poon et al. (2010), and Linder et al. (2007) who examined the association between EHR use and quality measures in ambulatory care settings but used different comparison groups for level of EHR use [15,17,25]. They compared EHR use to paper records, while this study compared it to partial/no EHR use. Given current EHR use requirements, there just were not enough offices with paper use only.

Further, we based our analysis on a theoretical framework by considering structure and process as the main components. Structure and process are essential because they can help predict outcomes of care delivered in physician offices. More details on the Donabedian models will be discussed in the following sections. Each quality measure will be discussed separately.

Diabetes measures

In this study, there were no correlations between EHR use and both diabetes measures (HbA1c and eye exam). It is noteworthy that the diabetes eye exam was borderline significant (negative association) in the unadjusted model. Contradicting our results, researchers found that EHR use improved diabetic individual measures (HbA1c and retinal exam) and composite compared to paper records [19]. Moreover, the rates of HbA1c testing were higher with EHR use in an ambulatory care setting, but diabetic eye exams did not show improvements [13]. The latter finding is consistent with our results. Similarly, when diabetic patients were followed for three years, EHRs did not ensure improving quality [20].

Several reasons can explain these contradictory findings. Cebul et al. (2011) used older data from NAMCS (2009-2010) to calculate composites of diabetic care [19]. Kern et al. (2013) compared EHRs to paper records in the Hudson Valley of New York, while this study compared all EHR use to partial/no use on a national level from more recent NAMCS data [13].

Obesity screening and education

Patients were more likely to receive obesity screening with all EHR use among visits to physician offices in both adjusted and unadjusted models. A study found that using EHRs improved BMI documentation, but no improvement in developing follow-up plans among overweight and obese patients [22]. Findings from that study confirmed our results. However, other researchers found that BMI documentation and obesity diagnosis were not always provided in EHR systems, which indicates either lack of documentation or an absence of screening [15,23]. Although this study found an association between EHR use and obesity screening, only 41% of visits had a documented BMI, implying that obesity screening is not always performed or recorded. Being diagnosed with obesity is a strong predictor of formulating a weight management follow-up plan. Therefore, it is crucial to improve obesity screening to prevent and control the obesity epidemic.

Romano and Stafford (2011) found that EHR use was related to improved diet and exercise counseling [24]. Their findings contradict our results; this might be due to the comparison categories of EHRs. They compared EHR use with paper records, while this study compared it with partial/no EHR use.

Breast cancer screening

Female patients were more likely to receive mammograms with all EHR use among visits to physician offices in both adjusted and unadjusted models. This is consistent with another study that found higher odds of breast cancer screening being associated with EHR use in a community-based setting [13]. In contrast, another study found that EHR use was not associated with improving women’s health, including breast cancer screening [15]. This contradiction could be attributed to the way Poon et al. (2010) examined outcomes as groups under women’s health or cancer prevention rather than breast cancer outcomes individually.

Depression screening

Contradicting our results, Akincigil and Matthews (2017) found that physicians who fully adapted EHRs were more likely to screen for depression when compared to physicians using paper records, even though depression screening was generally low [18]. Our findings also indicated that only 3.6% of eligible patients received depression screenings, which is considered fairly low. However, in our study, no correlation was found between depression screening and EHR use in ambulatory care. This discrepancy might be attributed to the different comparison groups.

Blood pressure screening

Patients were more likely to have blood pressure checked with all EHR use among visits to physician offices in both adjusted and unadjusted models. On the contrary, others found no difference or lower odds of blood pressure checks with any EHR use [17,24,25]. Although all three studies used NAMCS, they examined older data [25]. One explanation might be that EHR systems have advanced over time and/or medical professionals are now more accustomed to using these systems efficiently. It might also be due to the comparison categories of EHR use.

Structure and process

Researchers believe incorporating the Donabedian model in the analysis is helpful as structure and process components play vital roles in facilities’ capabilities and eventually can affect attributes and quality of care delivered. According to the Office of the National Coordinator for HIT, structure and process are commonly used quality measures in healthcare settings.

In our findings, no structure measures had a significant association with any quality measure except one negative association: physician offices in the western region had lower rates of depression screening. However, each individual process measure had a significant association with at least two quality measures, and, in some cases, with most quality measures, such as “primary care provider.” Previous literature did not focus on structure and process as main components. While some studies presented structure and process in crosstab results, no studies showed regression results. Therefore, our findings are a new addition to the literature. However, a systematic review used the structure and process components as outcomes in primary care and concluded that EHRs positively impacted both components compared to paper records. This research used the structure and process measurements as covariates (independent variables), while other studies used them as dependent variables [14].

Despite limited to no associations between structure or process and quality measures, the ROC analysis showed some interesting results. Because process measures already showed some significant results in the regression analysis, they showed the highest gain in AUC for all quality measures. Structure components showed a reasonable gain in AUC, especially for depression screening, despite no regression analysis associations. In regression models, each structural and process measurement is tested independently, reflecting minor changes in quality measures representing insignificant results. However, these variations are magnified when variables are grouped together, explaining the ROC results. AUC is commonly used when a large number of variables are studied and can be grouped together.

Limitations

The cross-sectional design of the study prevents inferring causality between variables. Secondary data analysis limited the ability to collect desired variables. As we were not able to include all components of the Donabedian model, analysis was restricted to structure and process but not health outcomes. NAMCS did not provide data related to patient outcomes such as morbidity and mortality. We wanted to include physician office size for structure measures, but the variable was masked in the dataset. Office size can significantly define the capabilities of the facility, as well as EHR capabilities. NAMCS response rate is considered relatively low for both years, which can cause nonresponse bias.

In addition, we did not control for patient compliance which might affect the rates of screening and counseling. Our analysis of visit records indicated if tests or services were performed, but no information was found related to physician’s orders that were not performed.

The selected quality measures only represent specific conditions and characteristics of care; therefore, it does not represent the overall care aspect. It is important to note that some associations may be caused by chance alone, and some associations are difficult to justify or explain. The perplexity and scarcity of these associations do not necessarily mean they are nonexistent. Furthermore, for a few quality measures, the small sample size may have prevented us from finding statistically significant associations.

## Conclusions

This research found positive associations between EHR use and better quality for three out of seven quality measures (obesity screening, blood pressure screening, and breast cancer). EHR might improve some clinical measures but not consistently improve all quality measures. We are not refuting that EHRs can improve quality in healthcare facilities in the United States, but findings suggest that as efforts to improve EHR use and adoption expand, automatic improvement of quality should not be assumed as a result. Moreover, measuring clinical quality should be continuously studied due to rapid advancements and changes in EHR systems over time. Evidence show improvement in some quality measures. Common and standardized health processes were more likely to be completed and recorded than others.

Policymakers are highly encouraged to consider focusing on future EHR criteria and efforts to improve prevention, screening, and counseling rates to facilitate quality care for populations. HIT future policies can shift the focus from improving EHR use to enhancing patient and quality outcomes. Future research should examine all three components of the Donabedian model to translate clinical quality outcomes (quality measures) to patient quality outcomes. Additionally, research should also focus on validating these findings on current EHR use because EHRs are advancing rapidly. Further research is needed to identify circumstances where quality is improved. Clinical decision support systems should be incorporated in future research to find details about associations between EHR and clinical quality measures. Clinical outcome improvement does not necessarily translate to patient outcomes, such as health improvements.

## References

[1] (2019). Clinical Quality Measures Basics. https://www.cms.gov/Regulations-and-Guidance/Legislation/EHRIncentivePrograms/ClinicalQualityMeasures.html.

[2] Collen MF, Ball MJ (2015). The History of Medical Informatics in the United States. https://link.springer.com/book/10.1007/978-1-4471-6732-7.

[3] (2020). Quick stats. https://dashboard.healthit.gov/quickstats/quickstats.php.

[4] (2020). Promoting interoperability (PI). https://www.cms.gov/Regulations-and-Guidance/Legislation/EHRIncentivePrograms/index.html.

[5] (2020). Certification of health IT. https://www.healthit.gov/topic/certification-ehrs/2015-edition.

[6] Casalino LP, Gans D, Weber R (2016). US physician practices spend more than $15.4 billion annually to report quality measures. Health Aff (Millwood).

[7] (2019). Data and program reports. https://www.cms.gov/Regulations-and-Guidance/Legislation/EHRIncentivePrograms/DataAndReports.html.

[8] Tang PC, McDonald CJ (2006). Electronic health record systems. Biomedical Informatics.

[9] Menachemi N, Collum TH (2011). Benefits and drawbacks of electronic health record systems. Risk Manag Healthc Policy.

[10] van Ginneken AM (2002). The computerized patient record: balancing effort and benefit. Int J Med Inform.

[11] Health IT (2020). Health IT. Benefits of EHRs. https://www.healthit.gov/topic/health-it-and-health-information-exchange-basics/benefits-ehrs.

[12] Campanella P, Lovato E, Marone C, Fallacara L, Mancuso A, Ricciardi W, Specchia ML (2016). The impact of electronic health records on healthcare quality: a systematic review and meta-analysis. Eur J Public Health.

[13] Kern LM, Barrón Y, Dhopeshwarkar RV, Edwards A, Kaushal R (2013). Electronic health records and ambulatory quality of care. J Gen Intern Med.

[14] Holroyd-Leduc JM, Lorenzetti D, Straus SE, Sykes L, Quan H (2011). The impact of the electronic medical record on structure, process, and outcomes within primary care: a systematic review of the evidence. J Am Med Inform Assoc.

[15] Poon EG, Wright A, Simon SR (2010). Relationship between use of electronic health record features and health care quality: results of a statewide survey. Med Care.

[16] Weigel FK, Switaj TL, Hamilton J (2015). Leveraging health information technology to improve quality in federal healthcare. US Army Med Dep J.

[17] Hsiao CJ, Marsteller JA, Simon AE (2014). Electronic medical record features and seven quality of care measures in physician offices. Am J Med Qual.

[18] Akincigil A, Matthews EB (2017). National rates and patterns of depression screening in primary care: results from 2012 and 2013. Psychiatr Serv.

[19] Cebul RD, Love TE, Jain AK, Hebert CJ (2011). Electronic health records and quality of diabetes care. N Engl J Med.

[20] Crosson JC, Ohman-Strickland PA, Cohen DJ, Clark EC, Crabtree BF (2012). Typical electronic health record use in primary care practices and the quality of diabetes care. Ann Fam Med.

[21] Howe RC, Murray M, Sharma S (2019). Five best practices for more effective use of ambulatory electronic health records to manage chronic disease. Tex Med.

[22] Gangadhar S, Nguyen N, Pesuit JW (2018). Effectiveness of a cloud-based EHR clinical decision support program for body mass index (BMI) screening and follow-up. AMIA Annu Symp Proc.

[23] Baer HJ, Karson AS, Soukup JR, Williams DH, Bates DW (2013). Documentation and diagnosis of overweight and obesity in electronic health records of adult primary care patients. JAMA Intern Med.

[24] Romano MJ, Stafford RS (2011). Electronic health records and clinical decision support systems: impact on national ambulatory care quality. Arch Intern Med.

[25] Linder JA, Ma J, Bates DW, Middleton B, Stafford RS (2007). Electronic health record use and the quality of ambulatory care in the United States. Arch Intern Med.

[26] Donabedian A (2005). Evaluating the quality of medical care. 1966. Milbank Q.

[27] Donabedian A (1988). The quality of care. How can it be assessed?. JAMA.

[28] (2019). Ambulatory health care data. https://www.cdc.gov/nchs/ahcd/about_ahcd.htm.

[29] (2020). Additional information regarding electronic clinical quality measures (eCQMs) for CMS quality reporting programs for eligible professionals and eligible clinicians. https://ecqi.healthit.gov/system/files/EP-EC-MeasuresTable-2018-05-v4.pdf.

[30] Hanley JA, McNeil BJ (1982). The meaning and use of the area under a receiver operating characteristic (ROC) curve. Radiology.

